# Long-Term Mortality and Causes of Death After Kidney and Liver Transplantation: A Nationwide Population-Based Cohort Study in South Korea

**DOI:** 10.3390/jcm15197446

**Published:** 2026-09-24

**Authors:** Eunjae Lee

**Affiliations:** Department of Nursing, U1 University, 310 Daehak-ro, Yeongdong-eup, Yeongdong-gun 29131, Republic of Korea; soa5070@naver.com

**Keywords:** kidney transplantation, liver transplantation, mortality, causes of death, standardized mortality ratio, cohort study

## Abstract

**Background/Objectives**: We analyzed long-term and cause-specific mortality among kidney and liver transplant recipients in South Korea. **Methods**: Using National Health Insurance Service and Statistics Korea data, we identified 24,332 kidney and 16,325 liver transplant recipients transplanted between 2006 and 2020. Three-, five-, and ten-year survival rates were estimated in nested fixed-entry subcohorts with observation closed at each horizon; multivariable Cox models were fitted in the full cohorts, with subcohort models used as consistency checks. Standardized mortality ratios (SMRs) applied identical cause-of-death classifications to observed and expected deaths (2011–2020). **Results**: Kidney recipient survival was 96.22% (95% CI 95.94–96.50), 94.53% (94.15–94.91), and 90.11% (89.31–90.92) at 3, 5, and 10 years, respectively; liver recipient survival was 81.28% (80.59–81.97), 78.27% (77.44–79.10), and 72.41% (71.02–73.80), respectively. Renal disease was the leading cause of death in kidney recipients (31.62%) and hepatic malignancy in liver recipients (42.35%). Male sex, older age, and lower income were associated with higher mortality in both cohorts; living-donor transplantation was associated with lower mortality than deceased-donor transplantation (hazard ratio [HR] 0.54; 0.50–0.58). Mortality was 3.65-fold and 7.58-fold that of the general population in kidney and liver recipients, respectively. Infection-related SMRs declined over time in both cohorts, whereas the suicide-related SMR among liver transplant recipients increased more than fourfold. **Conclusions**: Transplant recipients bear a substantially higher mortality burden than the general population, and the leading causes of death differ by transplanted organ and change over time. These findings support continued surveillance for malignancy, metabolic complications, and mental health.

## 1. Introduction

Life expectancy in South Korea increased from 80.6 years in 2011 to 83.5 years in 2020, while the prevalence of end-stage kidney disease and chronic liver failure also increased, imposing substantial health and economic burdens. Among solid-organ transplantation procedures, kidney and liver transplantation represent definitive treatments for end-stage organ failure and provide superior long-term survival and quality of life compared with dialysis or conservative treatment [1,2].

Advances in immunosuppressive therapy, surgical techniques, and postoperative care have markedly improved short-term graft and recipient survival. According to the Korea Network for Organ Sharing (KONOS), kidney and liver transplantation account for more than 80% of all solid-organ transplants in South Korea. The Korean transplant setting is characterized by the predominance of hepatitis B virus (HBV)-related liver disease as an indication for liver transplantation and a high proportion of living-donor liver transplantation, in contrast to Western countries, where hepatitis C and alcohol-related liver disease have traditionally been major indications [2,3].

Despite improvements in short-term survival, transplant recipients remain at substantially higher long-term mortality risk than the general population because of complications associated with immunosuppression, including opportunistic infection, de novo malignancy, metabolic disease, cardiovascular disease, and late allograft dysfunction [4,5]. The leading causes of death change over time: infection and rejection predominate early, whereas malignancy and cardiovascular disease become relatively more important beyond 5 years [5,6]. Socioeconomic disadvantage may also limit access to transplantation and worsen post-transplant outcomes [7].

Post-transplant outcome studies in South Korea have largely been limited to single centers, short follow-up periods, or specific complications [3,8]. The National Health Insurance Service (NHIS) database, which covers nearly the entire Korean population and is linked to Statistics Korea mortality data, provides a unique opportunity for representative long-term analyses [9]. Previous NHIS-based studies have examined graft survival after kidney transplantation and liver transplantation outcomes using registry data; however, no previous study has systematically compared cause-specific mortality and excess mortality relative to the general population across both organ types over a 15-year period [8,10].

Accordingly, this study aimed to comprehensively evaluate the epidemiology of post-transplant survival and mortality among kidney and liver transplant recipients using a multifaceted approach. We report all-cause and cause-specific mortality across 3-, 5-, and 10-year longitudinal observation windows and identify predictors of mortality to characterize risk profiles. In addition, standardized mortality ratios (SMRs) for 2011–2020 were used to compare cause-specific mortality among kidney and liver transplant recipients with that of the South Korean general population and to inform clinical care and health policy for transplant recipients.

## 2. Materials and Methods

### 2.1. Data Sources

This study used customized data from the National Health Insurance Service (NHIS), a mandatory single-payer health insurance system covering approximately 97% of the South Korean population. The NHIS database includes inpatient and outpatient claims, prescription records, health-screening results, insurance eligibility, and premium data. The structure and validity of the database have been described previously [9,11]. The customized extract supplied for this study covered claims from 2002 to 2020, and analyses were restricted to recipients who underwent transplantation between 2006 and 2020. The single underlying cause of death, coded using KCD-6 (equivalent to ICD-10), and date of death were obtained through linkage with Statistics Korea. Statistics Korea assigns one underlying cause of death per decedent according to WHO ICD-10 cause-of-death selection rules; multiple-cause and contributing-cause fields were not available, and every death in this study was therefore assigned to exactly one category. Annual age- and sex-specific death counts and mid-year population denominators for 2006–2020 were obtained from Statistics Korea; the SMRs themselves were computed for 2011–2020, as described in Section 2.4. The cause-of-death classification in Table 1 was applied identically to observed deaths in the cohorts and to the national cause-specific death counts used to derive expected deaths, so that the numerator and denominator of each cause-specific SMR shared the same definition.

### 2.2. Study Population

Kidney transplant recipients were identified using surgical procedure code R3280 together with a diagnosis code for kidney transplant status (Z94.0) or transplant failure/rejection (T86.1). After excluding records with missing income level (*n* = 491), residence (*n* = 5), or transplant year (*n* = 38), 24,332 kidney transplant recipients remained. Liver transplant recipients were identified using primary deceased- or living-donor transplant procedure codes Q8040–Q8050 together with Z94.4 or T86.4, excluding retransplantation (Q8140–Q8150). After applying the same missing-data exclusions (income, *n* = 384; residence, *n* = 10; transplant year, *n* = 38), 16,325 liver transplant recipients were included. Claims were retrieved only where the record itself carried one of the transplant diagnosis or procedure codes above, or a transplant-related special-exemption code: V005 and V013, which mark days on which immunosuppression was administered after kidney and liver transplantation, respectively, and V084 and V085, which identify registered kidney and liver transplant recipients. Accordingly, the extract represents transplant-related encounters rather than each recipient’s complete healthcare utilization, and covariates constructed from the extract should not be interpreted as resulting from a systematic search of the recipient’s complete medical history. Because the two case definitions were applied independently, a recipient of simultaneous liver–kidney transplantation satisfies both and is counted in each cohort; the total of 40,657 therefore refers to transplant episodes rather than to distinct individuals. The two cohorts were analyzed separately and were never combined. All transplantations were performed at transplant centers licensed under the Republic of Korea Internal Organs Transplant Act, and deceased-donor organs were allocated through the Korea Network for Organ Sharing; no organs from executed prisoners or from any commercial source were involved. Individual centers cannot be identified in the de-identified national claims data.

Follow-up ended on 31 December 2020. Recipients transplanted in later years could not accrue long observation periods, and a single pooled proportion would therefore combine patients observed for only a few months with those observed for as long as 15 years. To describe cause-of-death distributions over comparable periods, we defined three nested fixed-entry subcohorts that guaranteed at least 3, 5, or 10 years of potential follow-up from transplantation (transplanted in 2006–2017, 2006–2015, and 2006–2010, respectively). These subcohorts were intentionally overlapping rather than mutually exclusive. Recipients transplanted in 2006–2010 met all three entry criteria and therefore appeared in all three subcohorts. They were not independent groups intended for direct comparison but successive restrictions of the same cohort designed to show how the cause-of-death profile changed as the guaranteed observation window lengthened. Because the subcohorts were not defined as independent comparison groups, no between-subcohort statistical tests were performed. Differences between adjacent fixed-entry subcohort columns reflect not only the length of guaranteed observation but also the transplant era and case mix admitted by each entry window and should not be interpreted as isolated duration effects. Survival analyses were handled differently. Because right-censoring already accommodates unequal follow-up, restriction by transplant year would only reduce person-time and precision. Multivariable Cox models were fitted in the full cohorts and, as a consistency check, in each subcohort; subcohort estimates were generally consistent in direction with those from the full cohorts (Section 2.5). Accordingly, the full-cohort Cox models are presented as the primary analyses in Section 3.5 because they use all available person-time.

### 2.3. Variables and Definitions

Independent variables included sociodemographic factors (sex, age in 10-year groups, residence [urban/rural], and health-insurance premium quartile as a proxy for income [Q1 = lowest]) and clinical factors (primary underlying disease at transplantation, a dialysis-related diagnosis indicator in kidney transplant recipients, and donor type [living/deceased] in liver transplant recipients). The dialysis indicator, intended to capture pre-transplant dialysis, was ascertained from diagnosis codes rather than dialysis procedure claims and is therefore referred to throughout the manuscript as a “dialysis-related diagnosis.” Because data extraction was restricted to transplant-related encounters, nephrology visits or dialysis sessions without a transplant code were not retrieved. This variable therefore indicates whether a dialysis-related diagnosis happened to be coded on a claim already selected for a transplant code, most often the transplant admission, rather than reflecting a systematic search of each recipient’s pre-transplant history. It measures secondary diagnosis coding on a transplant-related encounter, and its observed prevalence depends more on coding practice and the composition of the retrieved claims than on the true frequency of pre-transplant dialysis. The primary underlying disease at transplantation was assigned from the principal diagnosis recorded on retrieved transplant-related claims. No ordered hierarchy was applied across disease categories, and secondary diagnosis fields were not used for disease assignment; therefore, recipients with multiple contributing diseases were represented by the condition recorded as the principal diagnosis. Cause-of-death categories were defined using KCD-6 code ranges (Table 1).

### 2.4. Standardized Mortality Ratio Estimation

SMRs were calculated as the number of observed deaths divided by the number of expected deaths. Expected deaths were estimated by multiplying annual age- and sex-specific mortality rates in the South Korean general population (Statistics Korea, 2011–2020) by the corresponding person-time of the transplant cohorts in each year. Age was stratified into five groups: <40, 40–49, 50–59, 60–69, and ≥70 years. An SMR > 1 indicated excess mortality relative to the general population.

### 2.5. Statistical Analysis

Descriptive statistics are presented as frequencies and percentages. Cause-specific proportional mortality was calculated as the proportion of deaths attributable to each cause among decedents. Survival from the date of transplantation was estimated using the Kaplan–Meier method, and differences between clinical subgroups over the full observation period were assessed using log-rank tests. Three-, five-, and ten-year survival rates were estimated in the corresponding fixed-entry subcohorts, with follow-up administratively closed at each horizon. Entry was restricted so that every recipient in each subcohort had at least the corresponding amount of potential follow-up; therefore, no recipient was censored before the horizon. Consequently, the Kaplan–Meier estimate at each horizon exactly equaled the observed survival proportion, and Greenwood variance reduced to the binomial variance, which was used to calculate the 95% confidence intervals reported in this study. Loss to follow-up other than death was not modeled. Because health insurance coverage is nearly universal and deaths were ascertained through national data linkage, such loss to follow-up is expected to be rare; however, recipients who left the insurance system would be counted as survivors, potentially biasing the estimates slightly upward. Multivariable Cox proportional hazards models were used to estimate hazard ratios (HRs) and 95% confidence intervals (CIs) for independent predictors of all-cause mortality, adjusted for transplant calendar year; model fit was assessed using likelihood-ratio tests. Cox models were fitted separately in the full kidney and liver cohorts and, as a consistency check, in each subcohort. Subcohort estimates were generally consistent in direction with full-cohort estimates, although some associations were no longer statistically significant in the smaller subcohorts. Only the full-cohort models are reported in the main text because they use all available person-time and therefore provide the most precise estimates. All analyses were performed using SAS version 9.4 (SAS Institute Inc., Cary, NC, USA), and two-sided *p* < 0.05 was considered statistically significant.

## 3. Results

### 3.1. Baseline Characteristics

Kidney transplant recipients (*n* = 24,332; Table 2) were predominantly male (59.85%), and the largest age group was 50–59 years (32.24%). Urban residents accounted for 70.85%. The income distribution was bimodal, with Q1 (31.43%) and Q4 (30.49%) each comprising approximately one-third of recipients. The most common underlying diseases were hypertension (36.83%), glomerular disease or unspecified chronic kidney disease (36.03%), and diabetes mellitus (21.98%). A dialysis-related diagnosis was recorded on a transplant-related encounter in 43.88% of recipients; this does not represent a measure of pre-transplant dialysis history. Mean follow-up was 2206 days.

Liver transplant recipients (*n* = 16,325; Table 3) were predominantly male (70.55%), and the largest age group was 50–59 years (43.16%). Living-donor transplantation accounted for 74.00% of procedures, consistent with Korean KOTRY registry data [10]. The most common underlying disease was hepatitis B (41.29%), followed by liver cancer (23.38%), advanced alcohol-related liver disease (15.63%), and hepatic steatosis (8.90%), a pattern consistent with the historically high burden of HBV-related liver disease in South Korea [12].

### 3.2. Overall Survival and Causes of Death

Among kidney transplant recipients, 1768 (7.27%) died during the full observation period. Survival was 96.22% at 3 years (95% CI: 95.94–96.50), 94.53% at 5 years (94.15–94.91), and 90.11% at 10 years (89.31–90.92) (Table 4). These estimates were similar to national KONOS survival rates of 94.5% at 5 years and 88.6% at 11 years [2] and to an NHIS-based report [8]. A death was classified as renal when the single underlying cause certified by Statistics Korea fell within the renal-disease code range listed in Table 1. This category therefore captures the terminal condition as certified on the death certificate but cannot distinguish graft failure with return to dialysis from native or recurrent renal disease, nor can it identify graft loss that occurred before death from another cause. Among certified underlying causes of death, renal disease was most common (31.62%), followed by malignancy (16.35%), metabolic disease (11.60%), cardiac disease (8.88%), and infection (5.71%). Suicide accounted for 2.21% of all deaths and 3.42% in the 10-year cohort.

Among liver transplant recipients, 3665 (22.45%) died during the full observation period. Survival was 81.28% at 3 years (95% CI: 80.59–81.97), 78.27% at 5 years (77.44–79.10), and 72.41% at 10 years (71.02–73.80) (Table 5). These estimates were also similar to national figures of 77.7% at 5 years and 71.4% at 11 years [2] and were consistent with Korean KOTRY data (5-year survival: LDLT 80.9%; DDLT 68.9%) [10]. Liver-related causes accounted for approximately 69% of all deaths. Hepatic malignancy was the most common cause (42.35%), followed by other liver disease (26.44%), infection (10.59%), and extrahepatic malignancy (6.33%). Suicide accounted for 0.96% of deaths in the 3-year cohort and 1.55% in the 10-year cohort.

### 3.3. Temporal Trends in Cause-Specific Mortality

Among kidney transplant recipients, the proportion of deaths attributed to renal disease declined from approximately 50% in 2006 to 25% in 2020, whereas malignancy increased to 22% and pulmonary disease to approximately 7%. These shifts mirror trends in the Australia and New Zealand Dialysis and Transplant Registry, where cancer deaths progressively approached cardiovascular deaths beyond the first post-transplant year [6]. Among liver transplant recipients, infection-related deaths declined from 32.56% in 2006 to 6.07% in 2020, a more than fivefold decrease. This decline was temporally compatible with improvements in antiviral and antimicrobial prophylaxis [3], but complete prophylaxis regimens could not be reconstructed from our extract and were not analyzed; this explanation therefore remains a hypothesis rather than a demonstrated mechanism. Hepatic malignancy remained the leading cause of death throughout the study period. Suicide among liver transplant recipients increased progressively, reaching 2.51% of all deaths in 2019.

### 3.4. Survival by Clinical Subgroup

Among kidney transplant recipients, underlying disease significantly predicted survival (log-rank *p* < 0.0001). Recipients with diabetes had a steeper decline in survival than those with hypertension or glomerular disease, with the curves separating clearly after approximately 3000 days post-transplant. The dialysis-related diagnosis indicator was associated with survival in univariable log-rank analysis (*p* = 0.0097) but was not an independent predictor in the multivariable Cox model; because this indicator does not represent pre-transplant dialysis history, no clinical interpretation was assigned to either finding. Among liver transplant recipients, hepatitis B as the underlying disease was associated with the most favorable long-term survival (log-rank *p* < 0.0001). Deceased-donor transplantation was associated with markedly higher mortality in the immediate postoperative period, consistent with KOTRY data reporting mortality of 14.7% among DDLT recipients and 4.0% among LDLT recipients [10].

### 3.5. Multivariable Risk Factors for Post-Transplant Mortality

Cox proportional hazards models were fitted in the full cohorts; results from the 3-, 5-, and 10-year subcohorts were generally consistent and are therefore not presented separately. Among kidney transplant recipients, male sex (HR 1.26; 95% CI: 1.14–1.39), older age, and lower income were independently associated with higher mortality (Table 6). With diabetes as the reference category, hypertension (HR 0.58; 95% CI: 0.50–0.63) and glomerular disease or unspecified chronic kidney disease (HR 0.54; 95% CI: 0.47–0.61) were associated with significantly lower mortality, whereas residence was not an independent predictor. The dialysis-related diagnosis indicator was also not independently associated with mortality (HR 1.01; 95% CI: 0.91–1.11). Because this indicator reflects diagnosis coding on transplant-related encounters rather than true dialysis history, the estimate cannot be interpreted as the effect of dialysis, and the direction of any resulting bias cannot be predicted. The estimate is therefore reported only for transparency regarding variables included in the fitted model. Overall findings were broadly similar to the USRDS-based analysis by Awan et al. [4], although the differences in cause-specific mortality proportions should be interpreted in light of the differences in cause-of-death classification discussed in Section 4.

Among liver transplant recipients, male sex (HR 1.22; 95% CI: 1.13–1.31), older age, and lower income independently predicted higher mortality (Table 7). Compared with hepatitis B, liver cancer (HR 1.31; 95% CI: 1.21–1.42), hepatic steatosis (HR 1.28; 95% CI: 1.14–1.45), and advanced alcohol-related liver disease (HR 1.19; 95% CI: 1.07–1.31) were associated with poorer prognosis. Living-donor transplantation was associated with substantially lower mortality (HR 0.54; 95% CI: 0.50–0.58), consistent with a meta-analysis of 39 studies (*n* = 38,563) showing significantly better 1-, 3-, and 5-year overall survival after LDLT than after DDLT [13].

### 3.6. Standardized Mortality Ratios

Across 2011–2020, kidney transplant recipients had a mean annual SMR of 3.65 (SD 0.43; range 2.96–4.20; Table 8). The highest cause-specific SMRs were for renal disease (mean 59.83) and metabolic disease (12.27). The renal-disease SMR declined from 100.34 in 2011 to 33.32 in 2020, and the infection SMR declined from 11.53 in 2011 to 4.12 in 2019. The malignancy SMR increased progressively, a direction consistent with the well-established excess cancer risk among transplant recipients [14]. However, for the reasons described in Section 4, cumulative immunosuppressive exposure could not be quantified in these data, and this trend cannot be directly attributed to cumulative immunosuppression.

Across 2011–2020, liver transplant recipients had a mean annual SMR of 7.58 (SD 1.90; range 4.76–10.70). The highest SMR was for digestive/liver disease (mean 96.05), reflecting the high proportion of hepatic malignancy. The infection SMR declined more than sixfold, from 60.06 in 2011 to 9.23 in 2020. As noted in the Table 1 footnote, the infection range includes chronic viral hepatitis (B16–B19); therefore, this category in the liver transplant cohort includes both conventional infection-related deaths and deaths in which the viral indication for transplantation itself was certified as the underlying cause. The decline in the infection-related SMR may therefore reflect changes in both opportunistic infections and hepatitis B-related deaths, but the contribution of these two components could not be distinguished in our data. The malignancy SMR decreased from 14.77 to 6.28. Cardiac disease had the lowest SMR (mean 1.58). The suicide SMR increased from 0.78 in 2011 to 3.15 in 2019, a more than fourfold increase over the period.

## 4. Discussion

This nationwide cohort study included 40,657 transplant episodes (24,332 kidney and 16,325 liver) identified in the NHIS database and followed for up to 15 years. Kidney and liver transplant recipients had markedly elevated overall mortality relative to the general population, with mean annual SMRs of 3.65 and 7.58, respectively. The leading causes of death differed substantially by transplanted organ and changed meaningfully over time. To our knowledge, this is the first population-based analysis in South Korea to simultaneously quantify cause-specific mortality, independent risk factors, and SMRs for both organ types using comprehensive national data.

The survival estimates in this study (kidney: 94.53% at 5 years and 90.11% at 10 years; liver: 78.27% and 72.41%, respectively) were very similar to KONOS registry statistics and Korean KOTRY data [2,10]. However, the leading causes of death differed substantially from those reported in Western cohorts. In a large study of 210,327 kidney transplant recipients in the United States (1996–2014), Awan et al. reported cardiovascular disease (24.7%) as the most common cause of death, followed by infection (15.2%) [4]. In our cohort, renal disease was most common (31.62%), followed by malignancy (16.35%). Part of this difference reflects differences in category construction rather than necessarily true epidemiologic differences and should be made explicit before substantive interpretation. Our cardiac-disease category includes ischemic, hypertensive, and other heart diseases but excludes cerebrovascular disease, which is classified as “Other” in our algorithm. By contrast, the USRDS-based classification used by Awan et al. includes cerebrovascular disease within cardiovascular mortality. Accordingly, our 8.88% and their 24.7% do not measure the same quantity, and the difference cannot be interpreted as evidence that Korean transplant recipients die less often from vascular causes. What can be said is that renal disease and malignancy accounted for a larger share of certified deaths in our cohort than in the US study. Beyond definitional differences, the greater frequency of hypertension and glomerular disease as principal diagnoses in Korean recipients, compared with diabetes as a leading diagnosis in US recipients, and the near-complete ascertainment of death through NHIS–Statistics Korea linkage may also have contributed to the observed differences, although these explanations were not tested. The increasing proportion of malignancy-related deaths over time is consistent with the established excess cancer risk among transplant recipients and is commonly attributed to cumulative immunosuppressive exposure [14]. We could not test this mechanism. As described in the limitations, the extract identifies days on which immunosuppression was administered but lacks dose and duration, precluding reconstruction of cumulative exposure. Thus, these data support the need for malignancy surveillance rather than a causal explanation.

Findings among liver transplant recipients were consistent with the NIDDK long-term follow-up study by Watt et al., which identified graft failure, malignancy, and cardiovascular disease as major late causes of death [5]. The particularly high proportion of hepatic malignancy in our cohort (42.35%) reflects the HBV-endemic setting of South Korea and the large contribution of HBV-related hepatocellular carcinoma to indications for liver transplantation [3,12]. Recipients with hepatitis B had the most favorable survival in both unadjusted and adjusted analyses, and understanding why is important when considering the generalizability of this finding. Since hepatitis B immune globulin combined with a high-barrier nucleos(t)ide analog became standard prophylaxis, HBV recurrence in the graft has become uncommon. Thus, the etiologic driver of the disease that brings many Korean patients to transplantation can be suppressed long-term after transplantation, unlike alcohol-related disease, in which exposure may recur, or steatotic liver disease, in which the metabolic substrate persists after transplantation and continues to drive cardiovascular and renal risks. In addition, HBV carriers in South Korea participate in hepatocellular-carcinoma surveillance, which may allow HBV-related transplant candidates to be detected within transplantable tumor limits and reach surgery earlier than patients whose liver disease presents late. Neither explanation could be tested in this study. The extract identifies the underlying-disease code but contains no viral load, HBeAg status, fibrosis stage, or tumor stage, and complete antiviral exposure, duration, and adherence could not be reconstructed from claims details alone. The survival advantage observed in the hepatitis B group should therefore be interpreted as an observation requiring confirmation in registries or cohorts with virologic and oncologic detail, not as evidence that HBV etiology is intrinsically protective. The decline in infection-related deaths from 32.56% to 6.07% is similar to trends in other national registries [5,6] and is compatible with improvements in antiviral and antimicrobial prophylaxis. However, prophylaxis exposure was not analyzed, and secular changes in death-certification practice, recipient selection, and underlying case mix may also explain part of the same trend.

Renal outcomes after liver transplantation warrant separate discussion because this is the area in which our data are most distant from the underlying clinical question. Renal disease accounted for only 1.26% of deaths in the liver transplant cohort, but this figure reflects only the single underlying cause selected by the certifying physician and does not measure the burden of renal dysfunction in these patients. Kidney injury in cirrhosis is heterogeneous. Hepatorenal syndrome–acute kidney injury (HRS-AKI) is a functional and potentially reversible state driven by splanchnic vasodilation, reduced effective arterial filling, and systemic inflammation, whereas acute tubular necrosis represents structural injury and is generally less responsive to volume expansion. Distinguishing these entities clinically is difficult and relies on creatinine kinetics, structured volume assessment, and tubular-injury biomarkers; the distinction affects both treatment and transplant strategy. Except when established end-stage kidney disease justifies simultaneous liver–kidney transplantation, isolated liver transplantation remains the default strategy [15]. These clinical constructs could not be reproduced in our data. Serum creatinine and estimated glomerular filtration rate were present in the extract for a subset of recipients who participated in the national health-screening program, so it would be inaccurate to say that no renal measurements existed; however, they were not used because they could not support the required distinction. Screening is periodic and unrelated in timing to episodes of acute kidney injury; participants differ systematically from nonparticipants and especially from the most severely ill patients, and values are unavailable at clinically critical times such as immediately before or after transplantation or death. Urine output, cystatin C, tubular-injury panels, and postoperative renal-function trajectories were also unavailable. We therefore could not distinguish a recipient with irreversible structural renal disease at transplantation from one who developed calcineurin-inhibitor nephrotoxicity years later, nor could we identify in whom renal function recovered after transplantation. Simultaneous liver–kidney transplantation could not be analyzed as a distinct exposure because no dedicated procedure code identified it during the study period, and such recipients met both case definitions. Accordingly, the renal-death estimate among liver transplant recipients should be interpreted as a floor rather than as an estimate of renal risk, and the absence of renal phenotyping should be understood as a boundary on the questions that a claims-based cohort can address rather than as a negative finding regarding the kidney after liver transplantation.

The survival advantage associated with living-donor transplantation (HR 0.54 among liver transplant recipients) is supported by a 2020 meta-analysis of 39 studies showing significantly better 1-, 3-, and 5-year overall survival after LDLT than after DDLT [13], as well as by KOTRY data in which mortality was 3.7-fold lower after LDLT [10]. Potential advantages include shorter cold ischemia time, better graft quality, and the ability to perform transplantation at an optimal time based on tumor biology. However, this estimate should be considered substantially confounded and was not interpreted causally. Donor type is not randomly assigned. Living donation requires a medically suitable and willing relative, and the recipient must be stable enough to await an elective operation, whereas deceased-donor grafts are preferentially allocated to the most urgent candidates. Our data lack measures of disease severity such as MELD score; transplant urgency, including waiting-list status or intensive care admission at surgery; tumor-burden variables such as Milan criteria, alpha-fetoprotein, and number and size of nodules; and donor or graft characteristics, including donor age, graft-to-recipient weight ratio, steatosis, cold ischemia time, and whole versus partial grafting. All of these factors may be related both to the probability of receiving a living-donor graft and to post-transplant survival and may therefore contribute to residual confounding. Nor can they be assumed to operate in the same direction; graft size and partial-graft factors in particular do not necessarily favor living donation. Thus, the adjusted HR of 0.54 represents a survival difference between two clinically dissimilar groups rather than the causal effect of donor type, the magnitude of which cannot be determined from these data.

The income gradient in post-transplant mortality is noteworthy. Because this study did not examine access to transplantation, the income disparity reported here is limited to post-transplant survival. A 2022 scoping review of 227 studies reported that socioeconomically disadvantaged patients were consistently less likely to receive transplant referral, evaluation, and wait-listing [7]. One plausible mechanism in the Korean setting is the out-of-pocket burden of lifelong immunosuppressive treatment, for which costs are only partially covered. However, this study did not measure out-of-pocket expenditure, medication adherence, co-payment exemption status, or supplemental private insurance, and the health insurance premium quartile is an imperfect proxy for household resources that also reflects employment status, age, and comorbidity. The reimbursement explanation is therefore a hypothesis generated by these data rather than a directly tested mechanism. The observed income disparity should be viewed as a signal for dedicated analyses linking insurance-premium data with pharmacy claims and adherence measures, rather than as direct evidence supporting a specific expansion of coverage.

The progressive increase in the suicide SMR among liver transplant recipients, from 0.78 in 2011 to 3.15 in 2019, is clinically important. A systematic review and meta-analysis by Dew et al. showed that depression and anxiety were independently associated with increased post-transplant morbidity and mortality [16], and depression is particularly common after liver transplantation, with a pooled prevalence of approximately 25% and the highest estimates reported in Asian populations [17]. Alcohol-related liver disease as a major indication for transplantation has often been suggested as a contributor to this pattern, but we did not test that association. The increase in the suicide SMR also coincided with a period in which national suicide reporting and death-certification practices were changing, so part of the increase may be nonclinical in origin. Our data identify the trend and its magnitude but cannot attribute it to any specific clinical factor. The annual numbers of suicide deaths underlying these ratios were small, limiting the precision of individual yearly estimates. Nevertheless, the direction of the trend was consistent in later years and supports attention to routine mental-health screening and integrated mental-health services in post-transplant follow-up.

This study has several limitations, most of which arise from the nature of the data source. First, and most importantly because it governs several subsequent limitations, data extraction was restricted to transplant-related encounters, as described in Section 2.2. Covariates constructed from the extract therefore describe what was coded on those encounters rather than a survey of each recipient’s complete medical history; the dialysis-related diagnosis indicator is the clearest example and is discussed separately in Section 2.3. Second, immunosuppressive exposure could not be quantified. The extract marks days on which post-transplant immunosuppression was administered through special-exemption codes used as part of the case definition, and ingredient codes are present in the claim detail, but daily dose, total days supplied, and drug-classification fields were not requested and are therefore unavailable. Consequently, cumulative exposure and immunosuppressive intensity could not be constructed, and we could not evaluate whether intensity differed by age group or mediated the observed cause-specific mortality patterns. Because immunosuppressive agents differ materially in nephrotoxic, metabolic, oncogenic, and infectious risks, this is a substantive rather than merely technical omission; a follow-up study designed around dispensing and prescription records would be a valuable extension. Third, the extract lacks measures of disease severity, transplant urgency, tumor burden, and graft function, so residual confounding cannot be excluded from any adjusted estimate. Laboratory values were available only as periodic results among a subset of recipients participating in the national health-screening program; their timing was unrelated to clinical events, and participants differed systematically from nonparticipants, making these measurements unsuitable for this purpose, and they were not analyzed. Fourth, no donor characteristics beyond living-versus-deceased status were available. Donor age, graft type, graft-to-recipient weight ratio, steatosis, and ischemia time were absent; moreover, among kidney transplant recipients, no dedicated code distinguished living from deceased donation during the study period, so donor type itself could not be identified. Fifth, cause of death was based on a single underlying cause selected on the death certificate, and inaccuracies in Korean death-certificate coding are well recognized [18]. Deaths resulting from interacting processes are assigned to only one category, so diseases acting as contributing factors rather than the terminal event are systematically underrepresented. Sixth, the cause-of-death algorithm shown in Table 1 was specified before analysis and has several features that should be considered when comparing our estimates with other studies. The cardiac-disease category excludes cerebrovascular disease and is therefore narrower than the composite cardiovascular categories used in most registries. The malignancy range includes in situ and benign neoplasms, although these are rarely certified as the underlying cause of death and were not excluded. The infection range includes rheumatic and other inflammatory cardiac codes and, in the liver transplant cohort, also includes chronic viral hepatitis, so infection-related mortality in that cohort does not represent conventional infectious deaths alone. These classification features are stated in the Table 1 footnote so that readers can judge their implications for any comparison of interest. Seventh, SMR estimates in the youngest age stratum (<40 years) were based on small numbers within a broad age category and should be interpreted cautiously. Eighth, the two organ cohorts were defined independently, so recipients of simultaneous liver–kidney transplantation contribute to both cohorts; the cohorts were analyzed separately and were never combined. Finally, as described in Section 2.5, the 3-, 5-, and 10-year survival estimates did not model loss to follow-up other than death. Recipients leaving the health-insurance system would be counted as survivors and could therefore bias survival slightly upward. Despite these limitations, nationwide coverage, linkage to national mortality data, and up to 15 years of follow-up allowed estimation of long-term cause-specific mortality in this population at a scale difficult to achieve in single-center studies.

## 5. Conclusions

Kidney and liver transplant recipients in South Korea experience substantially higher mortality than the general population, and the leading causes of death differ by transplanted organ and change markedly over time. Infection-related mortality has declined substantially, whereas malignancy, metabolic complications, and mental health—particularly suicide—are emerging as increasingly important challenges. These findings provide a basis for organ-specific surveillance programs, targeted interventions, and equitable policies aimed at improving long-term outcomes in this vulnerable population.

## Figures and Tables

**Table 1:** Definitions of independent and dependent variables (ICD-10/KCD-6 codes).

Category	Variable	Definition/ICD-10 (KCD-6) Code
Sociodemographic	Sex	Female; Male
	Age	<10, 10–19, 20–29, 30–39, 40–49, 50–59, 60–69, ≥70 years
	Residence	Urban (9 metropolitan cities and Gyeonggi Province); Rural (the remaining 9 provinces)
	Income level	National Health Insurance premium quartiles, Q1 (lowest)–Q4 (highest)
Underlying disease at kidney transplantation	Hypertension	I10–I15
	Diabetes mellitus	E10–E14
	Glomerular disease or unspecified chronic kidney disease	N00–N08 (glomerular disease); N18.9 (unspecified chronic kidney disease)
	Other	All diseases not listed above
Underlying disease at liver transplantation	Hepatitis B	B16.0–B16.2, B16.9, B18.0–B18.1
	Liver cancer	C22, C22.0–C22.9, C23, C24.0–C24.1, C24.8–C24.9
	Alcoholic hepatitis, fibrosis, cirrhosis, and hepatic failure	K70.10–K70.11, K70.2, K70.30–K70.31, K70.4, K70.42–K70.43, K70.9
	Hepatic steatosis and other non-necroinflammatory parenchymal liver disease	K70.0, K75.8, K76.0
	Hepatitis C	B18.2
	Other	All diseases not listed above
Clinical (kidney transplant only)	Dialysis-related diagnosis	Yes/No
Clinical (liver transplant only)	Donor type	Deceased donor (Q8040–Q8044); Living donor (Q8045–Q8050)
Cause of death (kidney)	Renal disease	N00–N06, N08, N10–N16, N17–N19, N20–N23, N28, I12, Q61
	Malignancy	C00–C97, D00–D36
	Metabolic disease	E00–E07, E10–E16, E20–E35, E50–E68, E70–E90
	Infection	A00–A09, A15–A19, A30–A32, A34–A49, A50–A79, B00–B09, B15–B34, B85–B99, I00–I02, I05–I09, I30–I36, I38, J12–J18, J20–J22, J30–J32, J36, J65, J67, J69, J85, K12
	Cardiac disease	I11, I20–I25, I26–I28, I37, I40–I52, I70–I79, I95–I99, R00–R01 (excluding cerebrovascular disease I60–I69)
	Pulmonary disease	J09–J11, J13–J18, J43–J47, J60–J70, J80–J86, J90–J99, R06
	Trauma	S00–S99, T00–T35, T67–T79, T90–T95, T98, V01–V99, W00–X59
	Suicide	X60–X84
Cause of death (liver)	Hepatobiliary malignancy	C22–C24
	Other liver disease	K70–K77, K80–K83, R16–R18, Q44
	Extrahepatic malignancy	C00–C97, D00–D36 (excluding C22–C24)
	Infection	Same ICD-10 range as in the kidney cohort
	Extrahepatic digestive disease	K20–K31, K35–K38, K40–K46, K50–K52, K55–K67, K85–K87, K90–K93, R11–R13, R15
	Cardiac disease, pulmonary disease, renal disease, trauma, suicide	Same as in the kidney cohort, with I10–I12 additionally included in cardiac disease

Note. ICD-10, International Classification of Diseases, 10th Revision; KCD-6, Korean Standard Classification of Diseases, 6th Revision. Cause-of-death categories were study-specific, and identical definitions were used for observed and expected deaths in SMR estimation. For overlapping codes, infection took precedence over pulmonary disease (J13–J18, J65, J67, J69, J85) and cardiac disease over renal disease in the liver cohort (I12). K70.0 was grouped with steatotic liver disease, whereas K70.1–K70.9 comprised advanced alcohol-related liver disease. Four features affect comparability with other series: cerebrovascular disease (I60–I69) is excluded from the cardiac category, which is therefore narrower than composite cardiovascular figures; in situ and benign neoplasms (D00–D36) are included in malignancy; rheumatic and cardiac inflammatory codes (I00–I02, I05–I09, I30–I36, I38) are included in infection; and chronic viral hepatitis (B15–B34) falls under infection rather than hepatic causes, so infection-related mortality in the liver cohort includes deaths certified to the transplant indication. Codes not listed were assigned to “Other”.

**Table 2:** Baseline characteristics of kidney transplant recipients (2006–2020).

Characteristic	Overall (2006–2020) *n* = 24,332	3-Year Cohort (2006–2017) *n* = 17,969	5-Year Cohort (2006–2015) *n* = 13,773	10-Year Cohort (2006–2010) *n* = 5321
Sex				
Female	9770 (40.15)	7304 (40.65)	5660 (41.09)	2294 (43.11)
Male	14,562 (59.85)	10,665 (59.35)	8113 (58.91)	3027 (56.89)
Age group (years)				
<10	122 (0.50)	94 (0.52)	76 (0.55)	40 (0.75)
10–19	436 (1.79)	347 (1.93)	305 (2.21)	163 (3.06)
20–29	1429 (5.87)	1148 (6.39)	978 (7.10)	504 (9.47)
30–39	3740 (15.37)	3093 (17.21)	2561 (18.59)	1201 (22.57)
40–49	6146 (25.26)	4787 (26.64)	3803 (27.61)	1569 (29.49)
50–59	7844 (32.24)	5721 (31.84)	4235 (30.75)	1394 (26.20)
60–69	4153 (17.07)	2542 (14.15)	1688 (12.26)	435 (8.18)
≥70	462 (1.90)	237 (1.32)	127 (0.92)	15 (0.28)
Income level, *n* (%)				
Q1 (lowest)	7647 (31.43)	6211 (34.57)	5007 (36.35)	2293 (43.09)
Q2	4172 (17.15)	3041 (16.92)	2383 (17.30)	965 (18.14)
Q3	5093 (20.93)	3770 (20.98)	2891 (20.99)	1158 (21.76)
Q4 (highest)	7420 (30.49)	4947 (27.53)	3492 (25.35)	905 (17.01)
Residence, *n* (%)				
Urban	17,240 (70.85)	12,807 (71.27)	9860 (71.59)	3821 (71.81)
Rural	7092 (29.15)	5162 (28.73)	3913 (28.41)	1500 (28.19)
Mean follow-up, days	2206	1068	1765	3452
Underlying disease at transplantation, *n* (%)				
Hypertension	8961 (36.83)	6613 (36.80)	5049 (36.66)	1962 (36.87)
Glomerular disease or unspecified CKD	8767 (36.03)	6912 (38.47)	5582 (40.53)	2411 (45.31)
Diabetes mellitus	5348 (21.98)	3576 (19.90)	2502 (18.17)	747 (14.04)
Other	1256 (5.16)	868 (4.83)	640 (4.65)	201 (3.78)
Dialysis-related diagnosis, *n* (%)				
No	13,655 (56.12)	10,733 (59.73)	9063 (65.80)	5234 (98.36)
Yes	10,677 (43.88)	7236 (40.27)	4710 (34.20)	87 (1.64)

Note. CKD, chronic kidney disease; Q, quartile based on National Health Insurance premiums.

**Table 3:** Baseline characteristics of liver transplant recipients (2006–2020).

Characteristic	Overall (2006–2020) *n* = 16,325	3-Year Cohort (2006–2017) *n* = 12,229	5-Year Cohort (2006–2015) *n* = 9581	10-Year Cohort (2006–2010) *n* = 3969
Sex, *n* (%)				
Female	4807 (29.45)	3497 (28.60)	2739 (28.59)	1130 (28.47)
Male	11,518 (70.55)	8732 (71.40)	6842 (71.41)	2839 (71.53)
Age group (years)				
<10	479 (2.93)	398 (3.25)	324 (3.38)	154 (3.88)
10–19	169 (1.04)	147 (1.20)	129 (1.35)	55 (1.39)
20–29	243 (1.49)	188 (1.54)	163 (1.70)	83 (2.09)
30–39	759 (4.65)	589 (4.82)	459 (4.79)	251 (6.32)
40–49	3060 (18.74)	2426 (19.84)	1987 (20.74)	974 (24.54)
50–59	7046 (43.16)	5458 (44.63)	4328 (45.17)	1727 (43.51)
60–69	4051 (24.81)	2712 (22.18)	2006 (20.94)	690 (17.38)
≥70	518 (3.17)	311 (2.54)	185 (1.93)	35 (0.88)
Income level, *n* (%)				
Q1 (lowest)	3906 (23.93)	3098 (25.33)	2478 (25.86)	1111 (27.99)
Q2	3119 (19.11)	2344 (19.17)	1859 (19.40)	811 (20.43)
Q3	3754 (23.00)	2817 (23.04)	2260 (23.59)	1018 (25.65)
Q4 (highest)	5546 (33.97)	3970 (32.46)	2984 (31.14)	1029 (25.93)
Residence, *n* (%)				
Urban	11,229 (68.78)	8464 (69.21)	6669 (69.61)	2773 (69.87)
Rural	5096 (31.22)	3765 (30.79)	2912 (30.39)	1196 (30.13)
Mean follow-up, days	1934	948	1533	2893
Underlying disease at transplantation, *n* (%)				
Hepatitis B	6741 (41.29)	5254 (42.96)	4236 (44.21)	1843 (46.43)
Liver cancer	3816 (23.38)	3142 (25.69)	2640 (27.55)	1355 (34.14)
Advanced alcohol-related liver disease	2552 (15.63)	1549 (12.67)	1006 (10.50)	190 (4.79)
Hepatic steatosis	1453 (8.90)	933 (7.63)	622 (6.49)	171 (4.31)
Hepatitis C	650 (3.98)	524 (4.28)	422 (4.40)	136 (3.43)
Other	1113 (6.82)	827 (6.76)	655 (6.84)	274 (6.90)
Donor type, *n* (%)				
Living donor	12,080 (74.00)	8951 (73.19)	7121 (74.32)	3145 (79.24)
Deceased donor	4245 (26.00)	3278 (26.81)	2460 (25.68)	824 (20.76)

**Table 4:** Survival and causes of death among kidney transplant recipients.

Outcome	Overall (2006–2020)	3-Year Cohort (2006–2017)	5-Year Cohort (2006–2015)	10-Year Cohort (2006–2010)
Survival, *n* (%)	22,564 (92.73)	17,290 (96.22)	13,019 (94.53)	4795 (90.11)
Death, *n* (%)	1768 (7.27)	679 (3.78)	754 (5.47)	526 (9.89)
Cause of death, *n* (% of decedents)				
Renal disease	559 (31.62)	287 (42.27)	294 (38.99)	191 (36.31)
Malignancy	289 (16.35)	63 (9.28)	103 (13.66)	84 (15.97)
Metabolic disease	205 (11.60)	88 (12.96)	104 (13.79)	66 (12.55)
Cardiac disease	157 (8.88)	47 (6.92)	52 (6.90)	39 (7.41)
Infection	101 (5.71)	38 (5.60)	38 (5.04)	24 (4.56)
Pulmonary disease	92 (5.20)	28 (4.12)	24 (3.18)	20 (3.80)
Trauma	46 (2.60)	13 (1.91)	17 (2.25)	12 (2.28)
Suicide	39 (2.21)	19 (2.80)	21 (2.79)	18 (3.42)
Other	280 (15.83)	96 (14.14)	101 (13.40)	72 (13.69)

Note. CI, confidence interval. Follow-up in the 3-, 5-, and 10-year fixed-entry subcohorts was administratively closed at each horizon. Because no censoring occurred before the horizon, the observed survival proportion equaled the Kaplan–Meier estimate. The corresponding 95% CIs were 95.94–96.50%, 94.15–94.91%, and 89.31–90.92%. The overall column represents the full follow-up period, not a fixed-time estimate.

**Table 5:** Survival and causes of death among liver transplant recipients.

Outcome	Overall (2006–2020)	3-Year Cohort (2006–2017)	5-Year Cohort (2006–2015)	10-Year Cohort (2006–2010)
Survival, *n* (%)	12,660 (77.55)	9940 (81.28)	7499 (78.27)	2874 (72.41)
Death, *n* (%)	3665 (22.45)	2289 (18.72)	2082 (21.73)	1095 (27.59)
Liver-related causes, *n* (% of decedents)				
Hepatic malignancy	1552 (42.35)	1073 (46.88)	1016 (48.80)	480 (43.84)
Other liver disease	969 (26.44)	615 (26.87)	468 (22.48)	212 (19.36)
Extrahepatic causes, *n* (% of decedents)				
Infection	388 (10.59)	299 (13.06)	271 (13.02)	185 (16.89)
Malignancy	232 (6.33)	72 (3.15)	105 (5.04)	67 (6.12)
Trauma	74 (2.02)	39 (1.70)	39 (1.87)	30 (2.74)
Pulmonary disease	69 (1.88)	21 (0.92)	25 (1.20)	12 (1.10)
Suicide	47 (1.28)	22 (0.96)	23 (1.10)	17 (1.55)
Cardiac disease	54 (1.47)	26 (1.14)	25 (1.20)	11 (1.00)
Renal disease	46 (1.26)	10 (0.44)	12 (0.58)	19 (1.74)
Digestive disease	32 (0.87)	13 (0.57)	10 (0.48)	10 (0.91)
Other	202 (5.51)	99 (4.33)	88 (4.23)	52 (4.75)

Note. CI, confidence interval. Follow-up in the 3-, 5-, and 10-year fixed-entry subcohorts was administratively closed at each horizon. Because no censoring occurred before the horizon, the observed survival proportion equaled the Kaplan–Meier estimate. The corresponding 95% CIs were 80.59–81.97%, 77.44–79.10%, and 71.02–73.80%. The overall column represents the full follow-up period, not a fixed-time estimate.

**Table 6:** Multivariable Cox proportional hazards analysis of factors associated with all-cause mortality among kidney transplant recipients (2006–2020).

Risk Factor	HR (95% CI)	*p*
Sex		
Female	1.00 (reference)	
Male	1.26 (1.14–1.39)	<0.001
Age (years)		
≥70	1.00 (reference)	
<10	0.05 (0.01–0.19)	<0.001
10–19	0.07 (0.04–0.13)	<0.001
20–29	0.05 (0.03–0.07)	<0.001
30–39	0.07 (0.06–0.10)	<0.001
40–49	0.13 (0.11–0.17)	<0.001
50–59	0.24 (0.19–0.29)	<0.001
60–69	0.51 (0.41–0.64)	<0.001
Income level		
Q1 (lowest)	1.00 (reference)	
Q2	0.80 (0.70–0.92)	<0.001
Q3	0.70 (0.61–0.80)	<0.001
Q4 (highest)	0.67 (0.59–0.76)	<0.001
Residence		
Rural	1.00 (reference)	
Urban	0.91 (0.82–1.01)	0.10
Underlying disease		
Diabetes mellitus	1.00 (reference)	
Hypertension	0.58 (0.50–0.63)	<0.001
Glomerular disease or unspecified CKD	0.54 (0.47–0.61)	<0.001
Other	0.56 (0.42–0.74)	<0.001
Dialysis-related diagnosis		
No	1.00 (reference)	
Yes	1.01 (0.91–1.11)	0.92

Note. CKD, chronic kidney disease; CI, confidence interval; HR, hazard ratio; Q, quartile based on National Health Insurance premiums. Estimates were obtained from multivariable Cox proportional hazards models adjusted for transplant calendar year.

**Table 7:** Multivariable Cox proportional hazards analysis of factors associated with all-cause mortality among liver transplant recipients (2006–2020).

Risk Factor	HR (95% CI)	*p*
Sex		
Female	1.00 (reference)	
Male	1.22 (1.13–1.31)	<0.001
Age (years)		
≥70	1.00 (reference)	
<10	0.26 (0.19–0.34)	<0.001
10–19	0.23 (0.15–0.36)	<0.001
20–29	0.38 (0.27–0.51)	<0.001
30–39	0.39 (0.31–0.48)	<0.001
40–49	0.42 (0.35–0.49)	<0.001
50–59	0.45 (0.38–0.52)	<0.001
60–69	0.57 (0.49–0.67)	<0.001
Income level		
Q1 (lowest)	1.00 (reference)	
Q2	0.90 (0.82–0.99)	0.03
Q3	0.87 (0.79–0.95)	<0.001
Q4 (highest)	0.86 (0.79–0.94)	<0.001
Residence		
Rural	1.00 (reference)	
Urban	1.00 (0.93–1.07)	0.89
Underlying disease		
Hepatitis B	1.00 (reference)	
Liver cancer	1.31 (1.21–1.42)	<0.001
Hepatitis C	1.17 (0.99–1.38)	0.07
Hepatic steatosis	1.28 (1.14–1.45)	<0.001
Advanced alcohol-related liver disease	1.19 (1.07–1.31)	<0.001
Other	1.03 (0.88–1.20)	0.74
Donor type		
Deceased donor	1.00 (reference)	
Living donor	0.54 (0.50–0.58)	<0.001

Note. CI, confidence interval; HR, hazard ratio; Q, quartile based on National Health Insurance premiums. Estimates were obtained from multivariable Cox proportional hazards models adjusted for transplant calendar year.

**Table 8:** Cause-specific standardized mortality ratios (SMRs) among kidney and liver transplant recipients compared with the South Korean general population (2011–2020).

Cause of Death	Mean SMR (SD)	Minimum SMR (Year)	Maximum SMR (Year)
Kidney transplant recipients			
Overall	3.65 (0.43)	2.96 (2014)	4.20 (2011)
Renal disease	59.83 (24.86)	33.32 (2020)	100.34 (2011)
Metabolic disease	12.27 (3.81)	7.45 (2019)	17.92 (2013)
Infection	7.81 (2.34)	4.12 (2019)	11.53 (2011)
Malignancy	4.52 (0.87)	3.21 (2011)	5.98 (2020)
Cardiac disease	2.08 (0.61)	1.24 (2019)	3.15 (2011)
Suicide	1.27 (0.36)	0.82 (2016)	1.89 (2019)
Liver transplant recipients			
Overall	7.58 (1.90)	4.76 (2020)	10.70 (2011)
Digestive disease (including liver disease)	96.05 (27.56)	58.40 (2020)	142.30 (2011)
Infection	26.73 (17.13)	9.23 (2020)	60.06 (2011)
Malignancy	10.84 (2.91)	6.28 (2020)	14.77 (2011)
Cardiac disease	1.58 (0.53)	0.91 (2017)	2.43 (2011)
Suicide	1.89 (0.89)	0.78 (2011)	3.15 (2019)

Note. SMR = observed deaths/expected deaths. Expected deaths were calculated using age- and sex-specific mortality rates for the South Korean general population (Statistics Korea). SD represents the standard deviation of annual SMR values over the 10-year period. Minimum and maximum SMRs and the corresponding years are shown in parentheses.

## Data Availability

The data analyzed in this study are available through the National Health Insurance Service (NHIS) Big Data Platform (https://nhiss.nhis.or.kr, accessed on 18 September 2026). Access is subject to NHIS review and approval, payment of applicable processing fees, and compliance with research ethics and data protection requirements. The author is not authorized to distribute these data directly to third parties.

## Abbreviations

The following abbreviations are used in this manuscript:

CI	Confidence interval
DDLT	Deceased-donor liver transplantation
HBV	Hepatitis B virus
HR	Hazard ratio
HRS-AKI	Hepatorenal syndrome–acute kidney injury
ICD-10	International Classification of Diseases, 10th Revision
KCD-6	Korean Standard Classification of Diseases, 6th Revision
KONOS	Korea Network for Organ Sharing
KOTRY	Korean Organ Transplantation Registry
LDLT	Living-donor liver transplantation
MELD	Model for End-Stage Liver Disease
NHIS	National Health Insurance Service
NIDDK	National Institute of Diabetes and Digestive and Kidney Diseases
SD	Standard deviation
SMR	Standardized mortality ratio
USRDS	United States Renal Data System
